# Exploring pain management preferences: a discrete choice experiment on cannabis or opioids among middle-aged and older adults

**DOI:** 10.1186/s41043-025-00989-x

**Published:** 2025-07-03

**Authors:** Rudiel Fabian, Paul Brown, Ricardo Cisneros

**Affiliations:** Department of Public Health, University of California Merced, 5200 N. Lake Road, Merced, CA 95348 USA

**Keywords:** Cannabis, Opioids, Over-the-counter medication, Discrete choice experiment, Policy options

## Abstract

**Objective:**

The study examines middle-aged and older adult’s preferences to identify policies that might increase the use of cannabis rather than opioids for controlling physical pain.

**Method:**

A discrete choice survey was administered to 301 older adults (≥ 40 years) in three California regions (Los Angeles, Bay Area, and San Joaquin Valley). Participants expressed preferences for pain medication under mild, moderate, and severe pain scenarios. Each participant made 16 choices between options that varied by medication type (medical cannabis, opioids, over-the-counter medications, or none), effectiveness, accessibility, side effects, addictiveness, and cost of the medication. A conditional logit model (CLM) was used to analyze the results for the entire sample, stratified by pain levels, and according to whether the participant had previous experience with taking medications for physical pain.

**Results:**

The results suggest that, all else equal, over-the-counter (OTC) treatments were the most preferred option for pain management. However, individuals were also willing to consider cannabis as a secondary treatment. Respondents with previous cannabis experience, either medically or recreationally, were more likely to select cannabis as their primary treatment choice. Marginal analysis revealed that the policy option of doubling opioid costs led to the greatest reduction in opioid use and increased the likelihood of cannabis use.

**Conclusions:**

Cannabis is a viable alternative to opioids for controlling pain. Findings suggest that interest in cannabis relative to opioids is high, and that messages that emphasize the addictiveness of opioids relative to cannabis might be particularly effective in decreasing opioid use.

## Introduction

Pain is defined as an unpleasant and emotional experience associated with actual or potential tissue damage [1]. It is a primary reason why adults seek medical care in the United States, highlighting its prevalence in the healthcare system [2]. According to the Centers for Disease Control and Prevention (CDC), approximately one in five adults in the U.S. experienced chronic pain in 2019, with one in fourteen experiencing ‘high impact’ chronic pain, defined as having pain on most days or every day during the past 3 months that limited life or work activities [3]. The incidence of physical pain increases with age, contributing to the millions of Americans who experience persistent pain annually.

 Chronic pain is typically managed through a combination of pharmacological and non-pharmacological approaches. Over-the-counter (OTC) medications such as acetaminophen and ibuprofen are commonly used as first-line treatments for mild to moderate pain, while prescription medications, including opioids, are often used for more severe or persistent cases [ 4 ]. Non-pharmacological options such as physical therapy, acupuncture, and chiropractic care are also widely employed as part of an integrated pain management strategy [ 5, 6 ]. Based on the prevalence of chronic pain, the total U.S. cost attributable to pain has been estimated at $560 to $635 billion, underscoring the economic burden of pain management [ 7 – 9 ].

Among pharmacological treatments, opioid use in older adults has emerged as a significant public health concern. In 2018, approximately 25% of adults aged 65 and older filled at least one opioid prescription [10]. Older adults are particularly susceptible to opioid-related harms, including increased risks of falls, cognitive impairment, respiratory depression, and dependence [11]. These risks are further compounded by age-related physiological changes, polypharmacy, commonly defined as the concurrent use of five or more medications and the presence of multiple chronic conditions [12]. Despite these concerns, opioids remain widely used for managing persistent pain in this population.

In response to the high cost, risks, and addictiveness of pain treatments such as opioids, there has been growing interest in alternative pain management options, including cannabis [13]. Evidence suggests that cannabis may offer therapeutic benefits for conditions such as chronic pain, multiple sclerosis, nausea, vomiting, cancer, post-traumatic stress disorder, epilepsy, and cachexia [14]. However, there are also adverse outcomes associated with recreational cannabis use, including motor vehicle accidents, psychosis, depression, suicide, and schizophrenia [15]. These risks are often influenced by factors such as dosage, frequency of use, and pre-exiting mental health conditions [15]. In clinical practice, most adverse events associated with medical cannabis use are mild and transient, primarily psychiatric symptoms (e.g., anxiety, mood changes) and nervous-system disturbances (e.g., dizziness, somnolence) underscoring the need for careful dose titration and ongoing monitoring [16].

Cannabis is a complex substance with multiple active ingredients, and the variability in its formulations have led to a lack standardized clinical guidelines leaving many clinicians unprepared to make evidence-based recommendations regarding its use for chronic pain management. Additionally, despite its medical potential cannabis remains federally prohibited in the United States. Nevertheless, as of 2023, 24 states have legalized cannabis for medical and recreational use [17]. In 1996, California became the first state to passed Proposition 215, which allowed patients and caregivers to present a physician’s recommendation as an affirmative defense against cannabis possession charges [18]. California later became the sixth state to legalize cannabis for recreational use in 2016 [19]. Since then, the accessibility of cannabis has significantly increased across various regions within the state, leading to a rapidly expanding retail market.

The purpose of this study is to examine older adult’s general attitudes towards using cannabis, opioids, OTC medication or no-treatment for physical pain management, and the effectiveness of policies that aim to increase the use of cannabis and decrease the use of opioids. While previous studies have indicated that individuals with pain conditions are turning to cannabis [13, 20], this study provides information on the willingness of older adults with general physical pain to consider cannabis as pain treatment option. A discrete choice experiment (DCE) was used to investigate the preferences and tradeoffs that older adults are willing to make concerning the access and use of cannabis for medical purposes, alongside the associated risks and benefits [20]. In addition, the results explore the likely impact of policies aimed to encourage cannabis use to control pain, including changing the relative price of each medication and informational campaigns focusing their relative addictiveness.

## Methods

A discrete choice experiment (DCE) is a quantitative method used to elicit preferences by presenting participants with hypothetical scenarios and asking them to choose between alternative options. DCEs are commonly applied in health economics and healthcare research to assess patient and provider preferences for different treatment attributes and outcomes. By analyzing these trade-offs, researchers can estimate the relative importance of various factors in decision-making and predict how individuals are likely to respond to changes in healthcare policies or treatment options [21].

The DCE design and experimental analysis followed the International Society for Pharmacoeconomics and Outcomes Research (ISPOR) checklist on patient-preference methods [22]. Specifically: (1) the identification of the key treatment attributes and assignment of levels to the attributes was done using interviews with older adults with experience in having chronic or persistent pain; (2) the experimental design evaluated alternative designs to optimize attribute combinations and choice tasks. The final survey design was selected based on maximizing efficiency score; (3) the treatment scenarios explored in the marginal analysis were constructed using various combinations of attributes and levels; and (4) the analysis of the choice data followed appropriate econometric procedures. Data is available from the authors by request.

### Development of attributes and levels

A two-stage process was followed to develop potential attributes. Phase one included a literature review of studies evaluating pain management, pain measurements, pain treatment options, including prescription medications, OTCs, and cannabis-based treatments [23–28]. The attributes were identified based on the most frequently reported factors influencing patient preferences in these studies. After careful consideration, 9 potential attributes (type of pain treatment [opioid, cannabis, OTC], effectiveness, ease of getting pain treatments, addictiveness/risk of dependence, side effects/adverse reactions, out-of-pocket cost, mode of administration/delivery method, doctor’s recommendation, and support [from families and friends]) [29–38] were derived from these studies and later consulted with an expert in the field. The levels for each attribute were then discussed, selected and defined with the expert in the field, resulting in 3–5 levels per attribute. The 9 attributes were then used in phase two of the process, which consisted of one-on-one interviews with 10 participants. Invitation letters for recruitment were created and distributed in the nearby downtown cannabis dispensary. A snowball sampling method was used in the process of reaching out to more participants willing to provide their personal experience with physical pain, and their use or knowledge of medical cannabis. It is important to note that participants did not need to have any prior experience with cannabis use. Those interested were emailed an invitation letter and a consent form. Most participants were Hispanic females; half were aged 18–35 and the other half were over 60, and the majority had prior cannabis experience (see Table 1). A saturation point was reached after 10 interviews.

**Table 1:** Demographic characteristics of interviewees

Interviewee	Sex/Gender	Race/Ethnicity	Age (years)	Prior Cannabis Experience
1	Female	Hispanic	62	Yes
2	Female	Hispanic	60	No
3	Female	White	66	Yes
4	Female	White	69	Yes
5	Male	White	35	Yes
6	Female	White	66	Yes
7	Female	Hispanic	34	No
8	Female	Hispanic	18	No
9	Male	Hispanic	35	Yes
10	Male	Hispanic	29	Yes

Each recorded interview was transcribed, cleaned and systematically coded in the Dedoose [39] software program, a qualitative analysis tool designed to facilitate the identification of themes and patterns within the interview data. The purpose of this qualitative phase was to gain a deeper understanding of how participants, both those with and without prior experience of physical pain and/or cannabis use for medical purposes prioritize different attributes when evaluating pain management options. By analyzing participant narratives, this process assessed the relative importance of the 9 initial attributes and their influence on treatment decision-making. Through this iterative process, the 9 attributes initially identified from the literature were refined based on participant responses, leading to the final selection of 7 key attributes that were deemed most relevant to decision-making in pain management. These final attributes include: (1) type of pain treatment, (2) pain severity before pain treatment, (3) pain severity after pain treatment (4) access/ease of getting pain treatments, (5) addictiveness, (6) side effects, and (7) cost (see Table 2).

**Table 2:** Attributes and levels

Attributes	Levels
Type of pain treatment	Over the counter medication
	Medical cannabis
	Opioid
	None
Pain severity before pain treatment	Mild (1 to 3 out of 10 on the pain scale)
	Moderate (4 to 7 out of 10 on the pain scale)
	Severe (8 to 10 out of 10 on the pain scale)
Pain severity after pain treatment	None (0 out of 10 on the pain scale)
	Mild (1 to 3 out of 10 on the pain scale)
	Moderate (4 to 7 out of 10 on the pain scale)
	Severe (8 to 10 out of 10 on the pain scale)
Access/ease of getting pain treatments	None
	Easy access
	Moderately difficult to access
	Hard to access
Addictiveness	Not at all addictive/able to quit at any time/no withdrawals or cravings
	Moderately addictive/would experience some withdraws or cravings when trying to quit
	Highly addictive/would be very difficult to quit/strong withdrawals and cravings
Side effects	Not at all or minimal (0% chance)
	Small probability of getting side effects (20% chance)
	Moderate probability of getting side effects (60% chance)
	High probability of getting side effects (90% chance)
Cost	No cost (free)
	$2.00 per treatment/$6.00 per day
	$5.00 per treatment/$15.00 per day
	$15.00 per treatment/$45.00 per day
	$25.00 per treatment/$75.00 per day

The *type of pain treatment* attribute was included to capture patient preferences for different treatment modalities, such as opioids, OTC medications, and medical cannabis, given their varying efficacy, side effect profiles, and accessibility. The *pain severity before and after treatment* were two essential attributes for assessing treatment effectiveness and capturing individual variations in pain perception and relief, which are key factors influencing treatment choice. *Access and ease of obtaining treatment* were included based on findings that logistical barriers such as prescription requirements, insurance coverage, and legal restrictions play a critical role in treatment selection and adherence. *Addictiveness* was identified as a crucial concern in both the literature and participant qualitative interviews, particularly regarding opioid-based treatments and, to some extent, cannabis. Patients weigh the potential risk of dependency when considering long-term pain management strategies. *Side effects* were incorporated as they directly impact treatment adherence and quality of life. In the qualitative interviews participants emphasized the trade-offs between efficacy and adverse effects, which often guide treatment preferences. Finally, *cost* emerged as a significant determinant in decision-making, as financial constraints often limit access to preferred treatments, particularly for individuals without adequate insurance coverage or those seeking alternative therapies like medical cannabis and heavily depending on out-of-pocket cost.

### DCE design

After final selection of the attributes and levels, the DCE choices were designed and validated using JMP Pro 17 software (SAS Institute Inc.). A full profile design was generated and randomized with all attributes and levels inserted accordingly, as seen on Table 2, the 7 attributes with 3–5 levels per attribute. After multiple test designs with a targeted sample size of 300, the best relative design was chosen based on the efficiency score (D-efficiency) of 96%, resulting in 8 versions and 16 choice sets per version. Data and information on the experimental design are available from the authors by request.

**Fig. 1:**
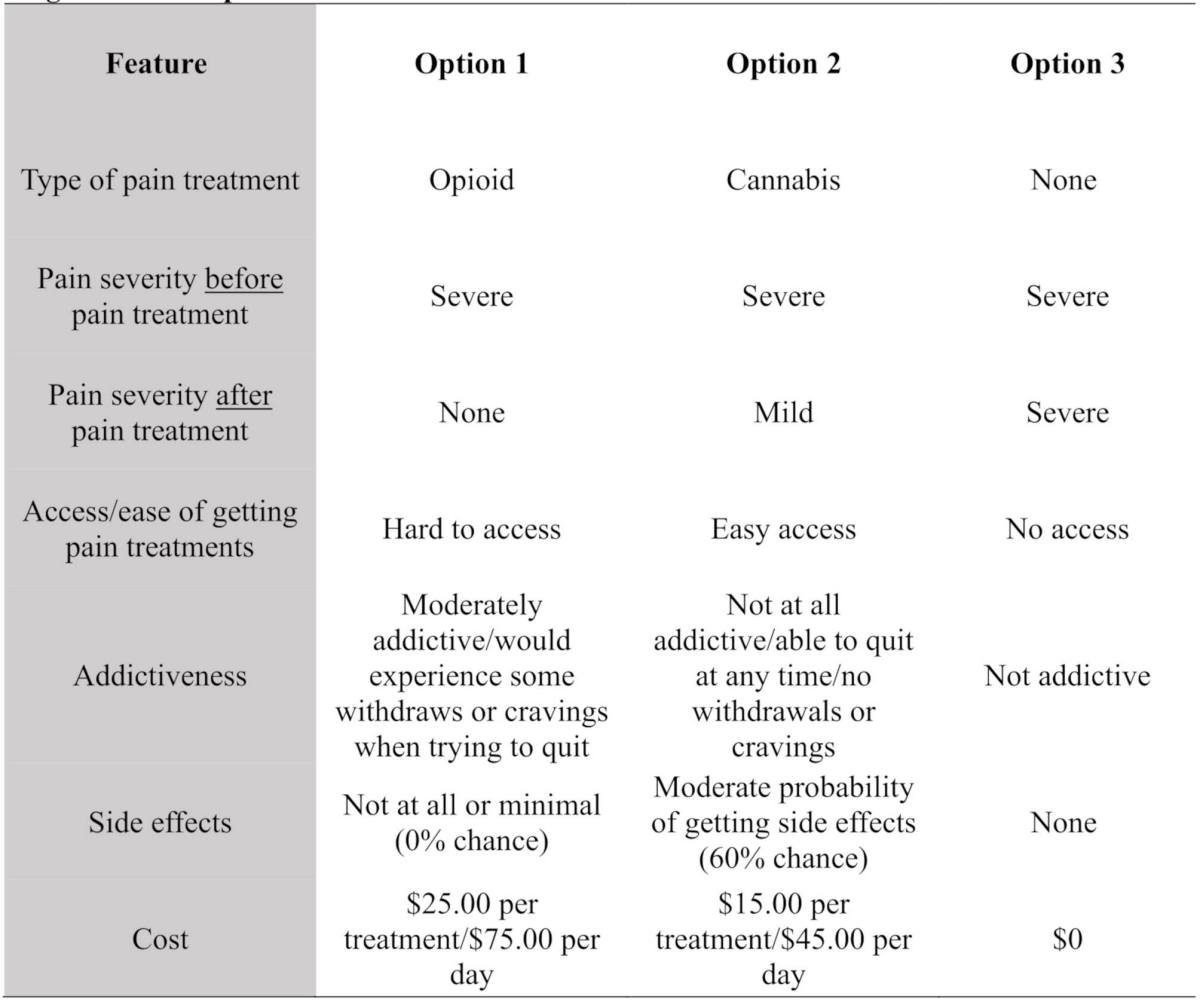
Example of a Choice Set with *Severe* Pain

Each choice set contained three choice options, the third choice being a “no-treatment” option and was included with the intention of offering a no-treatment alternative. However, values (levels) were given to this option, and all levels remained the same across all versions and choice sets within them, except under the “pain severity” attribute. While carefully evaluating the third-choice option, there was a high probability that participants will always choose ‘no pain’ after a pain treatment if they were given this level under Choice 3. Therefore, a decision was made to keep the ‘same’ pain severity before and after pain treatment based on the randomization from Choice 1 and 2. For example, if the “pain severity *before* pain treatment” was *severe* under Choice 1, then it remained *severe* on Choice 2 and *severe* on Choice 3, but it also remained *severe* on the “pain severity *after* pain treatment” attribute under Choice 3 only (see Fig. 1 for an example). It is also essential to mention that in Choice 1 and Choice 2, pain severity levels after treatment (e.g., none, mild, moderate, severe) varied by choice sets. Once the survey was completed and properly developed on the launch platform, it was then piloted and tested.

### DCE administration

Before completing the DCE section, participants were presented with a practice scenario that mimicked the purchasing of a vehicle (practice scenario is available from the authors upon request). Each vehicle choice had six attributes (color, make, miles, number of doors, model year and cost) with 2–3 levels each. Once familiarized with the format, they were taken to the administration of the DCE study where they were introduced to the 7 attributes and its levels, as previously presented in Table 2. Once on the DCE section, participants were assigned random versions in which they expressed their treatment preference (over the counter medication, medical cannabis, opioid or none) in 16 different scenarios with randomized levels and by choosing between 3 Choice options, Choice 3 always being a “no-treatment” option.

### Study sample

**Fig. 2:**
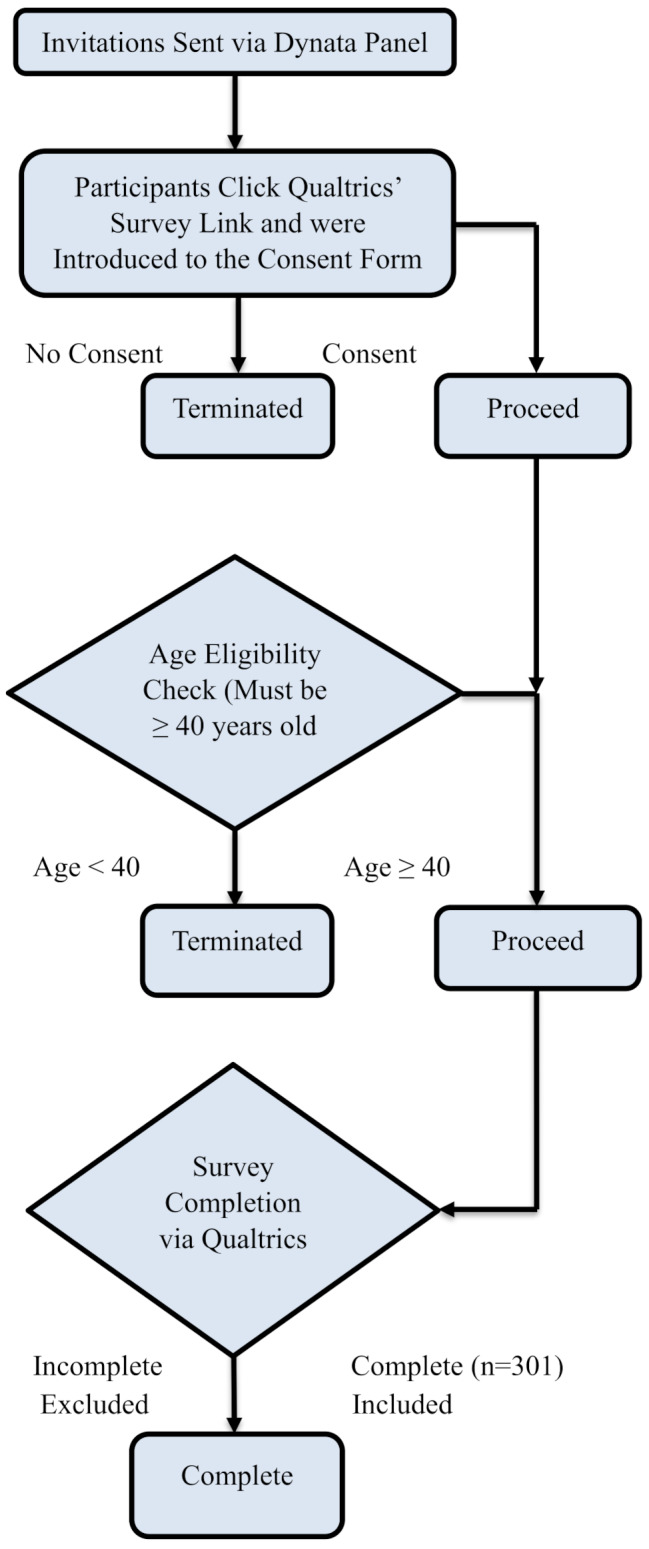
Recruitment Flowchart

An online survey was developed in the Qualtrics platform and conducted through Dynata [40] in December 2023 (*n* = 301). The survey targeted adults (≥ 40 years of age), encompassing both middle-aged (40–64 years) and older (65 + years) individuals. However, the entire sample in this study is referred to as “older adults” (see Fig. 2 for Recruitment Flowchart). The age quota was implemented to obtain a sample from older adults who are more likely to have experienced some type of physical pain in their life and have been exposed to different pain treatments. The survey also targeted three regions of the state, Los Angeles, the Bay Area, and the San Joaquin Valley. All participants were provided with a consent form and implied consent by taking the survey and were compensated upon completion by their panel provider. This study was approved by a university institutional review board.

### Measures

In addition to the DCE section, participants also responded to questionnaires on their experience with chronic pain, their previous experience with cannabis for recreational or medical use, their opinion on current cannabis policies and demographics.

#### Pain scale

To determine their pain severity experienced, participants were provided with a 10-point pain scale [41] and labeled mild, moderate, and severe [42]. Mild being from 1 to 3 out of 10, moderate being 4 to 7 out of 10, and severe being 8 to 10 out of 10 on the pain scale.

#### Cannabis use

As reported in previous studies, cannabis users (recreational or medical) were identified as having used cannabis within the last 30 days [43].

#### Region

Participants were categorized in three regions based on their collected zip code: Los Angeles, the Bay Area, and the San Joaquin Valley.

#### Demographics

All participants were asked to provide their age, 40 years old being the base year, gender, race/ethnicity, self-reported political views (liberal to conservative), income, marital status, and education level.

#### Perceptions of cannabis and opioids

All participants were asked to report their views of the cost, addictiveness, side effects, and ease of access to cannabis, opioids, and over-the-counter medications. These values were used in the base case in the marginal analysis.

### Data analysis

#### Discrete choice experiment

Descriptive statistics were used to characterize the demographics of the participants. For the discrete choice experiment (DCE) analysis, a conditional logit model (CLM) was used [44] in Stata 18, in which the dependent variable was the respondent’s choice for each hypothetical scenario, and the independent variables were the attribute levels. The first model included all the attributes as seen in Table 2, and in the second model, cost and side effects were linearized. The linearized models were used to compare the difference in preferences according to the three pain severity levels: Mild, Moderate, or Severe.

### Marginal analysis

Marginal probability is a powerful tool for modeling how consumers make decisions to maximize utility. The basic idea is that decision-makers make choices based on costs and benefits associated with small changes in a given state [45]. The marginal probabilities in this study were calculated using perceptions of the use of pain treatments. That is for the entire sample and for each group of pain severity levels (mild, moderate, and severe) separately. The probability of their perceptions (e.g., addictiveness, side effect, cost) was multiplied against the coefficient results from the linearized Conditional Logit Model (CLM) in the DCE and summed to estimate the utilities for each option. The marginal probabilities were then determined according to:

$$MP\left( {Ci} \right) = {{\exp \left( {{U_{Ci}}} \right)} \mathord{\left/{\vphantom {{\exp \left( {{U_{Ci}}} \right)} {\exp \left( {{U_{Ci}}} \right)}}} \right.\kern-\nulldelimiterspace} {\exp \left( {{U_{Ci}}} \right)}} + \exp \left( {{U_{Oi}}} \right)$$

where MP(C_i_) is the probability of choosing cannabis for group i, U_Ci_ is the utility associated with choosing cannabis for group i, and U_Oi_ is the utility associated with choosing opioid for group i. The probability of choosing opioid over cannabis for group i was 1– MP(_Ci_).

The marginal probabilities were calculated for several policy options aimed at promoting cannabis use. The base case was calculated using the strengths of preferences (coefficient values) from the DCEs (both combined for the entire sample and for each group separately) and the perceptions of each attribute (e.g., addictiveness, side effects, and cost associated with each treatment option). Thus, the base case represents the probability of choosing between cannabis and opioids given their perceptions of each treatment. The ‘base case’ to which these policies were compared used their perceived values of each of the different attributes, with the final utility being the weighted average of the responses:

$${U_{Ci}} = \sum {j\left( {\sum {k\left( {pro{b_k}*\beta k} \right)} } \right)} $$

where j are the attributes, prob_k_ is the percentage who choose option k for attribute j, and β_k_ is the coefficient from the discrete choice results for group i corresponding to option k of attribute j. The marginal analysis performed would encourage cannabis over opioids and based on their perceptions there are five policy options, the policy options explored in the marginal analysis were as follows:

- Doubling the price of opioid.
- Emphasizing high addictiveness of opioid.
- Lowering the price of cannabis by half.
- Emphasizing lower side effects of cannabis.
- Emphasizing lower addictiveness of cannabis.

## Results

### Sample

A sample of 301 Californian adults participated in the survey, aged 40 to 97 years with the mean age of 63.28 (± 13.93 years). The entire sample was evenly split, with females comprising (51%), mostly Non-Hispanic White (72%), with 4-year degree or higher (53%), identified themselves as extremely conservative (22%), and (45%) said to be retired at the time of survey. Participants were equally distributed across regions, from Los Angeles (33%), Bay Area (33%) and from the San Joaquin Valley (34%) (Table 3). Additionally, this distribution remained consistent between those who had experienced chronic pain and those who had not. Among participants with prior medical cannabis use, most were female (65%) and White (60%). In the prior opioid use group, a slight majority were female (54%) and White (85%). Participants who reported prior use of OTC medications or no treatment showed a sex and regional distribution similar to that of the overall sample.

**Table 3:** Demographic characteristics of study participants by chronic pain group and substance use experience %

	Total	Have not experienced chronic pain	Have experienced chronic pain	Prior Medical Cannabis Use	Prior Opioid Use	Prior OTC Medication or No Treatment
Sex/Gender						
Male	49%	59%	44%	35%	46%	43%
Female	51%	41%	56%	65%	54%	57%
Race/Ethnicity						
White	72%	67%	75%	60%	85%	74%
Hispanic	11%	11%	10%	15%	7%	11%
Black	5%	6%	4%	10%	0%	5%
Asian	8%	12%	5%	5%	2%	6%
Other	4%	4%	6%	10%	6%	4%
Education						
< 4-year degree	47%	36%	53%	60%	57%	50%
≥ 4-year degree	53%	64%	47%	40%	43%	50%
Conservatism						
Extremely	22%	22%	21%	20%	20%	22%
Age (in years)						
40–49	20%	18%	22%	45%	24%	17%
50–64	32%	27%	35%	40%	39%	32%
65+	48%	55%	43%	15%	37%	51%
Regions						
Los Angeles	33%	36%	32%	20%	30%	35%
Bay Area	33%	38%	30%	25%	26%	33%
San Joaquin Valley	34%	26%	38%	55%	44%	32%
N	301	112	189	20	54	115

### DCE results: entire sample

Results from the conditional analysis can be found in Table 4, and two separate models were included in this table. Model 1 provides the coefficient for each attribute assigned in this model. Model 2 reports the results after cost and side effects were linearized. After performing the first model, results indicated that the least preferred type of treatment was the “no treatment” option. In Model 1, the decision was to compare “no treatment” against every other type of treatment presented in Table 2, and the results on this model suggest that the most preferred type of treatment was “OTC” (β = 1.04, *P* < 0.001), followed by “cannabis” (β = 0.87, *P* < 0.001), and “opioid” (β = 0.61, *P* < 0.001) being the third most preferred method. The effectiveness of the treatment was measured in discrete step levels, where one design featured two sequential steps (from severe to mild or moderate to none), and another design featured three steps (from severe to none). By structuring improvement in this incremental manner, we captured how participants value varying degrees of pain relief. Ultimately, these levels “step2” (β = 0.24, *P* < 0.001) and “step3” (β = 0.59, *P* < 0.001) were the most preferred by participants. “Easy access” to treatments was also the most preferred method among them and significantly disliked “hard access” (β = -0.16, *P* < 0.01). In addition, individuals were also averse to treatments with “high side effects”, “highly addictive”, and with “higher cost”.

**Table 4:** Conditional and linearized models for all participants and by pain levels

Attributes and levels	Model 1	Model 2	Model 3		
	Conditional	Linearized	Mild	Moderate	Severe
	β(SE)	β (SE)	β (SE)	β (SE)	β (SE)
**Type of treatment**					
None	-	-	-	-	-
Over the counter (OTC)	1.04***	0.91***	0.30	0.73***	1.25***
	(0.08)	(0.07)	(0.23)	(0.11)	(0.10)
Cannabis	0.87***	0.74***	0.50	0.54***	1.05***
	(0.08)	(0.07)	(0.27)	(0.10)	(0.10)
Opioid	0.61***	0.49***	0.21	0.17	0.93***
	(0.09)	(0.08)	(0.24)	(0.13)	(0.13)
**Effectiveness**					
Step1	-	-	-	-	-
Step2	0.24***	0.26***		0.27***	0.16
	(0.05)	(0.05)		(0.08)	(0.09)
Step3	0.59***	0.58***			0.35***
	(0.07)	(0.07)			(0.09)
**Access to treatments**					
Easy	-	-	-	-	-
Moderate	− 0.06	-0.06	-0.18	0.05	-0.07
	(0.04)	(0.04)	(0.19)	(0.07)	(0.06)
Hard	-0.16*	-0.17**	-0.25	0.11	-0.29**
	(0.06)	(0.06)	(0.21)	(0.10)	(0.09)
**Addictiveness**					
None	-	-	-	-	-
Moderate	-0.33***	-0.33***	-0.46*	-0.36***	-0.29***
	(0.05)	(0.05)	(0.22)	(0.08)	(0.07)
High	-0.78***	-0.76***	-0.59*	-0.69***	-0.79***
	(0.06)	(0.06)	(0.26)	(0.09)	(0.09)
**Side effect**					
None	-				
Some chance (20%)	-0.14*				
	(0.06)				
Moderate chance (60%)	-0.22***				
	(0.05)				
High chance (90%)	-0.55***				
	(0.07)				
Linear	-	-0.01***	-0.00	-0.01***	-0.00***
	-	(0.00)	(0.00)	(0.00)	(0.00)
**Cost**					
No cost (free)	-				
$2.00	-0.27***				
	(0.07)				
$5.00	-0.39***				
	(0.07)				
$15.00	-0.76***				
	(0.06)				
$25.00	-1.14***				
	(0.08)				
Linear	-	-0.04***	-0.04***	-0.05***	-0.04***
	-	(0.00)	(0.01)	(0.01)	(0.00)

*p < 0.05; **p < 0.01; ***p < 0.001

### DCE results by pain severity levels

Additional linearized models were conducted by three different pain severity levels, mild, moderate, and severe (see Table 4). In the first model conducted by ‘mild’, only three predictors came out significant, participants disliked a “moderate” (β = -0.46, *P* < 0.01) and “highly addictive” (β = -0.59, *P* < 0.01), and “costly” (β = -0.04, *P* < 0.001) treatments, suggesting that with mild pain they do not have clear preferences. Under a ‘moderate’ level of pain, preferences changed significantly, having “OTC” (β = 0.73, *P* < 0.001) as the most preferred option, followed by “cannabis” (β = 0.54, *P* < 0.001). Participants also preferred a “step2” (β = 0.27, *P* < 0.001) effectiveness that would reduce the pain from moderate to none. They were also averse to treatments with “high side effects”, “moderate” and “highly addictive”, and with “higher cost”. For severe pain level almost all the predictors came out significant. At this level of pain, participants prefer all type of treatments provided as options, “OTC” (β = 1.25, *P* < 0.001), “cannabis” (β = 1.05, *P* < 0.001), and “opioid” (β = 0.93, *P* < 0.001). They also prefer a highly effective treatment, “step3” (β = 0.35, *P* < 0.001) that would reduce severe pain to none. In addition, the results also indicate a dislike for “hard access”, “moderate” and “highly addictive”, “high side effects”, and “costly” treatments.

### Perceptions of cannabis and opioids

 Table  5 reports the results on the perceptions of cannabis and opioids. Overall, participants reported to have easier access to cannabis over opioids. This could be explained by having legalized recreational cannabis use in the state of California in 2016, and most cities and counties have currently adopted some type of cannabis activities in their jurisdiction. Participants also reported to believe that they are less likely to get major side effects from cannabis use. From the entire sample, only (10%) reported cannabis to have ‘high’ side effects compared to opioids (43%). They also considered cannabis to be less addictive, only (15%) reported to consider it ‘highly’ addictive than opioids (71%). 50% believed that cannabis is less costly (less than $5) per treatment than opioids (24%). Similar results can be observed across groups where participants reported more favorable views towards cannabis than opioids.

**Table 5:** Perceptions of cannabis and opioids by pain severity levels %

	All		No Pain		Mild		Moderate		Severe	
	Cannabis	Opioid	Cannabis	Opioid	Cannabis	Opioid	Cannabis	Opioid	Cannabis	Opioid
Access										
Very difficult	6%	25%	8%	27%	9%	27%	10%	27%	12%	30%
Difficult	10%	36%	11%	44%	10%	42%	10%	45%	8%	50%
Neutral	26%	20%	30%	17%	29%	21%	28%	21%	22%	16%
Easy	34%	11%	33%	6%	34%	6%	33%	2%	34%	4%
Very Easy	23%	8%	18%	6%	18%	4%	19%	5%	24%	-
Side Effect										
Not at all (0%)	18%	3%	19%	3%	18%	3%	24%	-	24%	2%
Small (20%)	41%	11%	39%	11%	41%	11%	31%	10%	33%	8%
Moderate (60%)	31%	43%	29%	40%	29%	42%	26%	40%	22%	43%
High (90%)	10%	43%	13%	46%	12%	44%	19%	50%	21%	47%
Addictiveness										
Not at all	9%	3%	9%	5%	7%	4%	7%	5%	12%	8%
Low	38%	4%	37%	6%	40%	6%	40%	5%	38%	6%
Moderate	38%	22%	42%	19%	41%	21%	34%	17%	32%	15%
Highly	15%	71%	12%	70%	12%	69%	19%	73%	18%	71%
Cost										
$2 per treatment/$6 per day	9%	6%	7%	8%	6%	5%	10%	7%	10%	14%
$5 per treatment/$15 per day	41%	18%	41%	18%	41%	19%	33%	10%	39%	10%
$15 per treatment/$45 per	39%	37%	39%	29%	39%	29%	40%	22%	35%	23%
$25 or more per treatment/$75 per day	10%	39%	13%	45%	14%	47%	17%	61%	16%	53%

### Marginal analysis

The marginal analysis results suggest that all groups were sensitive to cost, side effects, and addictiveness. Table 6 shows the marginal probabilities for a choice between cannabis and opioids under a variety of conditions.

**Table 6:** Marginal probabilities by pain severity levels

Policies	All		Mild		Moderate		Severe	
	Cannabis	Opioid	Cannabis	Opioid	Cannabis	Opioid	Cannabis	Opioid
Base case	69%	31%	68%	32%	74%	26%	69%	31%
Double the cost of opioid	82%	18%	82%	18%	86%	14%	82%	18%
Emphasize addictiveness of opioid 90%	76%	24%	76%	24%	80%	20%	75%	25%
Lower the price of cannabis by half	74%	26%	73%	27%	79%	21%	74%	26%
Emphasize lower side effect of cannabis 20%	71%	29%	69%	31%	76%	24%	70%	30%
Emphasize lower addictiveness of cannabis 10%	75%	25%	75%	25%	79%	21%	73%	27%

For the base case analysis, the results suggest that the probability of choosing cannabis over opioids was 69% overall, including 68% among the ‘mild’ group, 74% among the ‘moderate’ group, and 69% among the ‘severe’ group. The impact of doubling the price of opioids, emphasizing the high addictiveness of opioids, lowering the price of cannabis by half from the initial base case, emphasizing lower side effects, and addictiveness of cannabis, the results suggest that these policies had a large impact on the behavior of its users, increasing the probability to use cannabis instead of opioids. Among the ‘all’ group and compared to the base case, doubling the price of opioids led to a decrease in the probability of its use by 13%. Among the ‘mild’ group, after the implementation of the same policy, opioids decreased by 14%, and the probability among the ‘moderate’ group decreased by 12%. The ‘severe’ group had a similar trend, the probability of opioid decreased by 13% respectively. Emphasizing the addictiveness of opioids to 90% among the ‘all’ group and compared to the base case reduces the probability of its use by 7%. Among the ‘mild’ group, the probability decreased by 8%, ‘moderate’ experienced a 6% decrease, and ‘severe’ had a 6% decrease as well.

Lowering the cost of cannabis by half compared to the base case, the probability of cannabis use among the ‘all’ group increased by 7%, the ‘mild’ group had a 5% increase, the ‘moderate’ experienced a 5%, and the ‘severe’ had a 5% increase as well. Emphasizing lower side effects of cannabis to 20% had a small impact among the ‘all’ group increasing the probability of using cannabis by only 2%, ‘mild’ increased by only 1%, ‘moderate’ increased by 2%, and ‘severe’ by only 1%. This means that opioid use would only have a relatively small impact reduction of 1% or 2% respectively. Emphasizing lower addictiveness of cannabis to 10%, the probability of its use among the ‘all’ group increased by 6%, ‘mild’ observed a 7% increase, ‘moderate’ had a 5% increase, and lastly, the ‘severe’ group had a 4% increase.

## Discussion

The purpose of this study was to examine older adults’ preferences for using opioids, cannabis, over-the-counter medications, or no treatment for managing physical pain. It also explored the potential effectiveness of policies aimed at increasing cannabis use and reducing opioid use. This focus on older adults is particularly important, as they are at elevated risk for both chronic pain and medication-related complications, especially due to age-related physiological changes and polypharmacy.

Historically, the management of chronic pain in the U.S. has relied heavily on opioids, despite their well-documented risks, including dependence, addiction, falls, cognitive impairment, and overdose [46, 47]. The findings reflect a shift away from opioids as the dominant modality. Participants showed a notable preference for OTC treatments, followed by cannabis, with opioids and “no treatment” being the least favored options.

Importantly, individuals with prior experience using medical or recreational cannabis were more inclined to prefer cannabis as their primary pain management option, suggesting that prior familiarity and comfort with cannabis could shape treatment preferences [48]. This is consistent with previous research indicating that patients with cannabis experience often report using it as a substitute for opioids due to its perceived effectiveness and more favorable side effect profile [49–51]. Moreover, the findings suggest that cost and risk perceptions significantly influence treatment selection. Specifically, interventions such as increasing the price of opioids and emphasizing their high addictiveness were associated with substantial shifts in preference toward cannabis. The sensitivity to cost is consistent with previous studies that have suggested that when financial and accessibility barriers to cannabis are minimized, individuals, particularly those with chronic pain, may choose cannabis over opioids [52–54].

The marginal analysis further reinforces the central finding that cost, addictiveness, and side effects significantly shape treatment preferences. Across all severity groups, mild, moderate, and severe, participants showed a consistent and substantial shift toward cannabis when opioid costs were doubled, with a 13% decrease in the probability of choosing opioids in the overall sample. Similarly, emphasizing the high addictiveness of opioids reduced the likelihood of their selection by 7% overall. These patterns are consistent with earlier studies showing that economic and risk-related messaging can meaningfully alter pain management choices [52, 55]. Reductions in the cost of cannabis and messaging around its lower addictiveness also led to meaningful increases in its selection, particularly among the mild and moderate groups. Although emphasizing cannabis’s lower side effects had only modest changes, this may reflect a ceiling effect in perceptions of cannabis safety among current users or those already favoring non-opioid options. These findings support prior research suggesting that structural levers such as pricing, public health campaigns, and coverage policies can be used to promote safer alternatives to opioids, especially for populations managing chronic pain [56, 57]. The policy scenarios in this section were selected to represent the most feasible interventions corresponding to the attributes evaluated in the DCE. Policymakers should consider the relative responsiveness to these levers across subgroups, particularly as interventions to reduce opioid-related harms continue to evolve.

Cannabis users in this study also prioritized treatment effectiveness, aligning with previous research indicating that individuals often favor cannabis for its pain-relieving properties and lower incidence of adverse effects compared to opioids [58–61]. This was particularly evident among participants with moderate or severe pain, where cannabis and OTCs were consistently rated higher than opioids in terms of desirability. Studies have consistently found that experienced cannabis users are more likely to return to cannabis for pain management, often citing better symptom control and tolerable side effects [62–64]. These findings suggest that cannabis may be appealing as a non-pharmaceutical alternative for individuals seeking to manage pain while minimizing harm.

While these findings highlight cannabis’s growing role in pain management, the study also has limitations. First, qualitative interviews with 10 participants were conducted to validate and prioritize attributes identified from the literature. The primary aim of these interviews was attribute confirmation rather than discovery; while it is possible that older males might identify additional attributes not captured here, no substantial gender differences emerged in how participants, male or female, responded to the prioritization questions. Because all five older-adult interviewees were female, perspectives unique to older men remain unexamined, but the consistency across genders suggests limited impact on the final attribute set. Second, the sample for discrete choice experiment (DCE) was drawn from an online panel and may not fully represent the broader population, particularly with respect to ethnic diversity among California residents. Notably, some subgroups with historically higher rates of cannabis use may be underrepresented. Third, although DCEs offer a powerful tool to model decision-making, they are inherently based on hypothetical scenarios and may not capture the full complexity of real-world treatment decisions. Fourth, focusing solely on pharmacologic treatments allowed for a streamlined DCE design, reducing attributes, levels and respondent burden, but excluded nonpharmacological options (e.g., physical therapy, acupuncture). This may overemphasize drug-related factors (cost, efficacy, side effects, addictiveness) and underplay trade-offs with non-medication strategies. Future DCE studies should incorporate both pharmacologic and nonpharmacologic modalities to fully capture patient preferences in chronic pain management. Fifth, geographic variation in treatment access such as differences by ZIP code or region was not modeled in this study but represents an important area for future exploration. Nevertheless, understanding consumer preferences remains essential for guiding policies that reflect public attitudes and promote safer pain management alternatives.

Another potential limitation is that participants were not required to have personal experience using cannabis or opioids. However, this design choice was deliberate. We included individuals with and without such experience to assess how lived experience influences preferences and to explore how the general public, especially those who may newly experience pain, perceive their treatment options. This approach is important not only for comparing preference patterns but also for informing public health and policy decisions. Many regulatory shifts around cannabis and opioid access require broad public support, including from individuals who may not yet have personal experience with chronic pain but could in the future. Capturing these perspectives provides insight into potential future patient behavior and broader societal attitudes toward alternative pain management strategies. Moreover, participants were also asked about their prior use of opioids and cannabis to allow for subgroup comparisons based on lived experience.

This study underscores the importance of considering both patient preference and real-world feasibility in developing policies that address the opioid crisis through alternative treatments. By modeling the tradeoffs older adults are willing to make, the results provide empirical evidence to guide public health campaigns, insurance coverage decisions, and regulatory reforms. As the accessibility of cannabis continues to expand and interest in alternatives to opioids grows, understanding the nuanced preferences of older adults, both with and without prior cannabis experience will be critical in shaping effective, equitable, and responsive pain management strategies. These insights are especially pertinent for regions considering cannabis legalization, or for healthcare systems reevaluating the role of cannabis in chronic pain care.

## Data Availability

No datasets were generated or analysed during the current study.

## References

[1] Treede RD, The International Association for the Study of Pain Definition of Pain. As valid in 2018 as in 1979, but in need of regularly updated footnotes. PAIN Rep. 2018;3(2). 10.1097/pr9.0000000000000643 10.1097/PR9.0000000000000643 PMC5902252 29756089

[2] Schappert SM, Burt CW. Ambulatory care visits to physician offices, hospital outpatient departments, and emergency departments: United States, 2001–02. Vital Health Stat 13. 2006;(159):1–66. Available from: https://stacks.cdc.gov/view/cdc/6677 16471269

[3] Centers for Disease Control and Prevention. CDC Clinical Practice Guideline for prescribing opioids for pain - United States, 2022. November 3, 2022. 10.15585/mmwr.rr7103a1

[4] Chou R, Deyo R, Friedly J, et al. Systemic Pharmacologic therapies for low back pain: a systematic review for an American college of physicians clinical practice guideline. Ann Intern Med. 2017;166(7):480–92. 10.7326/M16-2458 28192790 10.7326/M16-2458

[5] Qaseem A, Wilt TJ, McLean RM, Forciea MA. Noninvasive treatments for acute, subacute, and chronic low back pain: a clinical practice guideline from the American college of physicians. Ann Intern Med. 2017;166(7):514–30. 10.7326/M16-2367 28192789 10.7326/M16-2367

[6] Skelly AC, Chou R, Dettori JR et al. Noninvasive Nonpharmacological Treatment for Chronic Pain: A Systematic Review Update. Agency for Healthcare Research and Quality (US); 2020. Available from: https://www.ncbi.nlm.nih.gov/books/NBK556223/32338846

[7] Gaskin DJ, Richard P. The economic costs of pain in the united States. J Pain. 2012;13(8):715–24. 10.1016/j.jpain.2012.03.009 22607834 10.1016/j.jpain.2012.03.009

[8] Gaskin DJ, Richard P, Walburn J. The economical impact of pain. In: Saba L, editor. Neuroimaging of pain. Cham: Springer; 2017. 10.1007/978-3-319-48046-6_1

[9] Schirmer D, Karri J, Abd-Elsayed A. Economic burden of pain. In: Abd-Elsayed A, editor. Guide to the inpatient pain consult. Cham: Springer; 2020. 10.1007/978-3-030-40449-9_37

[10] Centers for Disease Control and Prevention (CDC). Opioid Prescribing and Use in Older Adults — United States, 2018. MMWR Morb Mortal Wkly Rep. 2020;69(11):298–302. Available from: https://www.cdc.gov/mmwr/volumes/69/wr/mm6911a5.htm 10.15585/mmwr.mm6911a5 PMC7739983 32191686

[11] Virnes R-E, Tiihonen M, Karttunen N, et al. Opioids and falls risk in older adults: A narrative review. Drugs Aging. 2022;39(3):199–207. 10.1007/s40266-022-00929-y 35288864 10.1007/s40266-022-00929-y PMC8934763

[12] Varghese D, Ishida C, Patel P, Koya HH, Polypharmacy. StatPearls Publishing. 2024. Available from: https://www.ncbi.nlm.nih.gov/books/NBK532953/30422548

[13] Shi Y, Cao Y, Shang C, Pacula RL. The impacts of potency, warning messages, and price on preferences for cannabis flower products. Int J Drug Policy. 2019;74:1–10. 10.1016/j.drugpo.2019.07.037 31382201 10.1016/j.drugpo.2019.07.037 PMC6893125

[14] National Academies of Sciences, Engineering, and Medicine. The health effects of cannabis and cannabinoids: the current state of evidence and recommendations for research. Washington, DC: National Academies; 2017. 10.17226/24625 28182367

[15] Centers for Disease Control and Prevention. What we know about marijuana. March 1. 2023. https://www.cdc.gov/marijuana/featured-topics/what-we-know-about-marijuana.html

[16] Crescioli G, Lombardi N, Bettiol A, et al. Adverse events following cannabis for medical use in tuscany: an analysis of the Italian phytovigilance database. Br J Clin Pharmacol. 2020;86(1):106–20. 10.1111/bcp.14140 31656045 10.1111/bcp.14140 PMC6983517

[17] Aletraris L, Graves BD, Ndung’u JJ. Assessing the impact of recreational cannabis legalization on cannabis use disorder and admissions to treatment in the united States. Curr Addict Rep. 2023;10(2):198–209. 10.1007/s40429-023-00470-x 37266190 10.1007/s40429-023-00470-x PMC10088679

[18] Pacula RL, Smart R. Medical marijuana and marijuana legalization. Annu Rev Clin Psychol. 2017;13:397–419. 10.1146/annurev-clinpsy-032816-045128 28482686 10.1146/annurev-clinpsy-032816-045128 PMC6358421

[19] Orenstein DG, Glantz SA. The grassroots of grass: Cannabis legalization ballot initiative campaign contributions and outcomes, 2004–2016. J Health Polit Policy Law. 2019;45(1):73–109. 10.1215/03616878-7893579 10.1215/03616878-7893579 PMC6980940 31675092

[20] Wilson L, Zheng P, Ionova Y, et al. Caper: patient preferences to inform nonsurgical treatment of chronic low back pain: A discrete-choice experiment. Pain Med. 2023;24(8):963–73. 10.1093/pm/pnad038 36975607 10.1093/pm/pnad038 PMC12394812

[21] Bridges JFP, Hauber AB, Marshall D, et al. Conjoint analysis applications in health—a checklist: a report of the ISPOR good research practices for conjoint analysis task force. Value Health. 2011;14(4):403–13. 10.1016/j.jval.2010.11.013 21669364 10.1016/j.jval.2010.11.013

[22] Bridges JFP, de Bekker-Grob EW, Hauber B, et al. A roadmap for increasing the usefulness and impact of patient-preference studies in decision making in health: A good practices report of an ISPOR task force. Value Health. 2023;26(2):153–62. 10.1016/j.jval.2022.12.004 36754539 10.1016/j.jval.2022.12.004

[23] Häuser W, Petzke F, Fitzcharles MA. Efficacy, tolerability, and safety of cannabis-based medicines for chronic pain management—An overview of systematic reviews. Eur J Pain. 2018;22(3):455–70. 10.1002/ejp.1118 29034533 10.1002/ejp.1118

[24] Krebs EE, Gravely A, Nugent S, et al. Effect of opioid vs nonopioid medications on pain-related function in patients with chronic back pain or hip or knee osteoarthritis pain: the SPACE randomized clinical trial. JAMA. 2018;319(9):872–82. 10.1001/jama.2018.0899 29509867 10.1001/jama.2018.0899 PMC5885909

[25] Penney LS, Ritenbaugh C, DeBar LL, et al. Provider and patient perspectives on opioids and alternative treatments for managing chronic pain: a qualitative study. BMC Fam Pract. 2016;17(1):164. 10.1186/s12875-016-0566-0 10.1186/s12875-016-0566-0 PMC5390355 28403822

[26] Vowles KE, McEntee ML, Julnes PS, et al. Rates of opioid misuse, abuse, and addiction in chronic pain: a systematic review and data synthesis. Pain. 2015;156(4):569–76. 10.1097/01.j.pain.0000460357.01998.f1 25785523 10.1097/01.j.pain.0000460357.01998.f1

[27] Moore RA, Derry S, Aldington D, et al. Adverse events associated with single dose oral analgesics for acute postoperative pain in adults-an overview of Cochrane reviews. BMJ. 2015;350:h3196. 10.1002/14651858.CD011407.pub2 26461263 10.1002/14651858.CD011407.pub2 PMC6485338

[28] Sites BD, Harrison J, Herrick MD, et al. Prescription opioid use and satisfaction with care among adults with musculoskeletal conditions. Ann Fam Med. 2018;16(1):6–13. 10.1370/afm.2148 29311169 10.1370/afm.2148 PMC5758314

[29] Sullivan MD, Howe CQ. Opioid therapy for chronic pain in the united states: promises and perils. Pain. 2013;154(Suppl 1):S94–100. 10.1016/j.pain.2013.09.009 24036286 10.1016/j.pain.2013.09.009 PMC4204477

[30] Nugent ST, Veerabagu SA, Madden M, et al. Patient preferences for pain control after Mohs micrographic surgery: A single-center discrete choice experiment. JAMA Dermatol. 2023;159(9):1085–92. 10.1001/jamadermatol.2023.1899 37405725 10.1001/jamadermatol.2023.1899 PMC10323759

[31] Krebs EE, Carey TS, Weinberger M. Accuracy of the pain numeric rating scale as a screening test in primary care. J Gen Intern Med. 2007;22(10):1453–8. 10.1007/s11606-007-0321-2 17668269 10.1007/s11606-007-0321-2 PMC2305860

[32] Farrar JT, Young JP, LaMoreaux L, et al. Clinical importance of changes in chronic pain intensity measured on an 11-point numerical pain rating scale. Pain. 2001;94(2):149–58. 10.1016/s0304-3959(01)00349-9 11690728 10.1016/S0304-3959(01)00349-9

[33] Hachem Y, Abdallah SJ, Rueda S, et al. Perceptions and barriers of healthcare professionals toward medical cannabis use for cancer pain management. Cannabis Cannabinoid Res. 2022;7(6):764–71. 10.1186/s12906-022-03716-9

[34] Goshua A, Craigie S, Guyatt GH, et al. Patient values and preferences regarding opioids for chronic noncancer pain: A systematic review. CMAJ. 2018;190(27):E792–800. 10.1093/pm/pnx274 10.1093/pm/pnx274 29618109

[35] Labianca R, Sarzi-Puttini P, Zuccaro SM, et al. Adverse effects associated with non-opioid and opioid treatment in patients with chronic pain. Clin Drug Investig. 2012;Suppl 153–63. 10.2165/11630070-000000000-00000

[36] Dunphy C, Peterson C, Zhang K, et al. Do out-of-pocket costs influence retention and adherence to medications for opioid use disorder? Drug Alcohol Depend. 2021. 10.1016/j.drugalcdep.2021.108784 34049104 10.1016/j.drugalcdep.2021.108784 PMC8314254

[37] Donnan JR, Johnston K, Coombs M, et al. Exploring consumer preferences for cannabis edible products to support public health policy: A discrete choice experiment. PLoS ONE. 2024. 10.1371/journal.pone.0292336 38753807 10.1371/journal.pone.0292336 PMC11098505

[38] Bentley C, Izadi-Najafabadi S, Raymakers A, et al. Qualitative research informing a preference study on selecting cannabis for cancer survivor symptom management: design of a discrete choice experiment. BMC Public Health. 2022;22(1):1125. 10.1007/s40271-021-00567-3 35132605 10.1007/s40271-021-00567-3 PMC9197893

[39] SocioCultural R, Consultants LLC. Dedoose (Version 10.0.23) [Computer software]. Los Angeles, CA: SocioCultural Research Consultants, LLC; 2023. Available from: https://www.dedoose.com/

[40] Dynata LLC. Dynata [Internet]. Shelton, CT. Available from: https://www.dynata.com/

[41] The pain of measuring pain. Harvard Health. December 1. 2018. https://www.health.harvard.edu/pain/the-pain-of-measuring-pain

[42] Von Korff M, DeBar LL, Krebs EE, et al. Graded chronic pain scale revised: mild, bothersome, and high-impact chronic pain. Pain. 2019;161(3):651–61. 10.1097/j.pain.0000000000001758 10.1097/j.pain.0000000000001758 PMC7097879 31764390

[43] Centers for Disease Control and Prevention. Exposure to advertisements and marijuana use among US adolescents. November 30, 2017. https://www.cdc.gov/pcd/issues/2017/17_0253.htm

[44] Hauber AB, González JM, Groothuis-Oudshoorn CGM, et al. Statistical methods for the analysis of discrete choice experiments: A report of the ISPOR conjoint analysis good research practices task force. Value Health. 2016;19(4):300–15. 10.1016/j.jval.2016.04.004 27325321 10.1016/j.jval.2016.04.004

[45] McFadden D. Economic choices. Am Econ Rev. 2001;91(3):351–78. 10.1257/aer.91.3.351

[46] Haley DF, Saitz R. The opioid epidemic during the COVID-19 pandemic. JAMA. 2020;324(16):1615. 10.1001/jama.2020.18543 32945831 10.1001/jama.2020.18543

[47] Bernard SA, Chelminski PR, Ives TJ, Ranapurwala SI. Management of pain in the united States—a brief history and implications for the opioid epidemic. Health Serv Insights. 2018;11. 10.1177/1178632918819440 10.1177/1178632918819440 PMC6311547 30626997

[48] Boehnke KF, Scott JR, Litinas E, et al. Cannabis use preferences and decision-making among a cross-sectional cohort of medical cannabis patients with chronic pain. J Pain. 2019;20(11):1362–72. 10.1016/j.jpain.2019.05.009 31132510 10.1016/j.jpain.2019.05.009

[49] Lucas P, Walsh Z. Medical cannabis access, use, and substitution for prescription opioids and other substances: A survey of authorized medical cannabis patients. Int J Drug Policy. 2017;42:30–5. 10.1016/j.drugpo.2017.01.011 28189912 10.1016/j.drugpo.2017.01.011

[50] Piper BJ, DeKeuster RM, Beals ML, et al. Substitution of medical cannabis for pharmaceutical agents for pain, anxiety, and sleep. J Psychopharmacol. 2017;31(5):569–75. 10.1177/0269881117699616 28372506 10.1177/0269881117699616

[51] Reiman A, Welty M, Solomon P. Cannabis as a substitute for opioid-based pain medication: patient self-report. Cannabis Cannabinoid Res. 2017;2(1):160–6. 10.1089/can.2017.0012 28861516 10.1089/can.2017.0012 PMC5569620

[52] Haroutounian S, Ratz Y, Ginosar Y, et al. The effect of medicinal cannabis on pain and quality-of-life outcomes in chronic pain: A prospective open-label study. Clin J Pain. 2016;32(12):1036–43. 10.1097/ajp.0000000000000364 26889611 10.1097/AJP.0000000000000364

[53] Bar-Lev Schleider L, Mechoulam R, Lederman V, et al. Prospective analysis of safety and efficacy of medical cannabis in large unselected population of patients with cancer. Eur J Intern Med. 2018;49:37–43. 10.1016/j.ejim.2018.01.023 29482741 10.1016/j.ejim.2018.01.023

[54] Abuhasira R, Schleider LBL, Mechoulam R, Novack V. Epidemiological characteristics, safety and efficacy of medical cannabis in the elderly. Eur J Intern Med. 2018;49:44–50. 10.1016/j.ejim.2018.01.019 29398248 10.1016/j.ejim.2018.01.019

[55] Bachhuber MA, Saloner B, Cunningham CO, Barry CL. Medical cannabis laws and opioid analgesic overdose mortality in the united states, 1999–2010. JAMA Intern Med. 2014;174(10):1668–73. 10.1001/jamainternmed.2014.4005 25154332 10.1001/jamainternmed.2014.4005 PMC4392651

[56] Bradford AC, Bradford WD, Abraham A, Bagwell Adams G. Association between US state medical cannabis laws and opioid prescribing in the medicare part D population. JAMA Intern Med. 2018;178(5):667–72. 10.1001/jamainternmed.2018.0266 29610897 10.1001/jamainternmed.2018.0266 PMC6145794

[57] Wen H, Hockenberry JM. Association of medical and adult-use marijuana laws with opioid prescribing for medicaid enrollees. JAMA Intern Med. 2018;178(5):673–9. 10.1001/jamainternmed.2018.1007 29610827 10.1001/jamainternmed.2018.1007 PMC6145792

[58] Kruger DJ, Kruger JS, Collins RL. Cannabis enthusiasts’ knowledge of medical treatment effectiveness and increased risks from cannabis use. Am J Health Promot. 2020;34(4):436–9. 10.1177/0890117119899218 31916839 10.1177/0890117119899218

[59] Bonn-Miller MO, Boden MT, Bucossi MM, Babson KA. Self-reported cannabis use characteristics, patterns and helpfulness among medical cannabis users. Am J Drug Alcohol Abuse. 2013;40(1):23–30. 10.3109/00952990.2013.821477 24205805 10.3109/00952990.2013.821477

[60] Jennings JM, Johnson RM, Brady AC, Dennis DA. Patient perception regarding potential effectiveness of cannabis for pain management. J Arthroplasty. 2020;35(12):3524–7. 10.1016/j.arth.2020.06.051 32684396 10.1016/j.arth.2020.06.051

[61] Li X, Vigil JM, Stith SS, et al. The effectiveness of self-directed medical cannabis treatment for pain. Complement Ther Med. 2019;46:123–30. 10.1016/j.ctim.2019.07.022 31519268 10.1016/j.ctim.2019.07.022

[62] Piper BJ, Beals ML, Abess AT, et al. Chronic pain patients’ perspectives of medical Cannabis. Pain. 2017;158(7):1373–9. 10.1097/j.pain.0000000000000899 28328576 10.1097/j.pain.0000000000000899 PMC5845915

[63] Corroon J, Mischley L, Sexton M. Cannabis as a substitute for prescription drugs-a cross-sectional study. J Pain Res. 2017;10:989–98. 10.2147/jpr.s134330 28496355 10.2147/JPR.S134330 PMC5422566

[64] Sexton M, Cuttler C, Finnell JS, Mischley LK. A cross-sectional survey of medical cannabis users: patterns of use and perceived efficacy. Cannabis Cannabinoid Res. 2016;1(1):131–8. 10.1089/can.2016.0007 28861489 10.1089/can.2016.0007 PMC5549439

